# The Simplified Nutritional Appetite Questionnaire (SNAQ) as a Screening Tool for Risk of Malnutrition: Optimal Cutoff, Factor Structure, and Validation in Healthy Community-Dwelling Older Adults

**DOI:** 10.3390/nu12092885

**Published:** 2020-09-21

**Authors:** Sabrina Lau, Kalene Pek, Justin Chew, Jun Pei Lim, Noor Hafizah Ismail, Yew Yoong Ding, Matteo Cesari, Wee Shiong Lim

**Affiliations:** 1 Department of Geriatric Medicine, Tan Tock Seng Hospital, Singapore 308433, Singapore; Justin_CHEW@ttsh.com.sg (J.C.); Jun_Pei_LIM@ttsh.com.sg (J.P.L.); yew_yoong_ding@ttsh.com.sg (Y.Y.D.); wee_shiong_lim@ttsh.com.sg (W.S.L.); 2 Institute of Geriatrics and Active Ageing, Tan Tock Seng Hospital, Singapore 308433, Singapore; Kalene_SL_PEK@ttsh.com.sg (K.P.); noor_hafizah@ttsh.com.sg (N.H.I.); 3 Department of Continuing and Community Care, Tan Tock Seng Hospital, Singapore 308433, Singapore; 4 Department of Clinical Sciences and Community Health, University of Milan, 20122 Milan, Italy; macesari@gmail.com; 5 Geriatric Unit, IRCCS Istituti Clinici Scientifici Maugeri, 20122 Milan, Italy

**Keywords:** malnutrition, anorexia, older adults, cutoff, factor structure, validity

## Abstract

Malnutrition is an independent marker of adverse outcomes in older adults. While the Simplified Nutritional Appetite Questionnaire (SNAQ) for anorexia has been validated as a nutritional screening tool, its optimal cutoff and validity in healthy older adults is unclear. This study aims to determine the optimal cutoff for SNAQ in healthy community-dwelling older adults, and to examine its factor structure and validity. We studied 230 community-dwelling older adults (mean age 67.2 years) who were nonfrail (defined by Fatigue, Resistance, Ambulation, Illnesses & Loss (FRAIL) criteria). When compared against the risk of malnutrition using the Mini Nutritional Assessment (MNA), the optimal cutoff for SNAQ was ≤15 (area under receiver operating characteristic (ROC) curve: 0.706, sensitivity: 69.2%, specificity: 61.3%). Using exploratory factor analysis, we found a two-factor structure (Factor 1: Appetite Perception; Factor 2: Satiety and Intake) which accounted for 61.5% variance. SNAQ showed good convergent, discriminant and concurrent validity. In logistic regression adjusted for age, gender, education and MNA, SNAQ ≤15 was significantly associated with social frailty, unlike SNAQ ≤4 (odds ratio (OR) 1.99, *p* = 0.025 vs. OR 1.05, *p* = 0.890). Our study validates a higher cutoff of ≤15 to increase sensitivity of SNAQ for anorexia detection as a marker of malnutrition risk in healthy community-dwelling older adults, and explicates a novel two-factor structure which warrants further research.

## 1. Introduction

Malnutrition is increasingly recognized as an important and independent marker of adverse outcomes in older adults, including higher chronic disease burden, frailty and mortality [1,2,3]. The process of malnutrition and involuntary weight loss can be driven by anorexia, inadequate dietary intake, sarcopenia, cachexia, or a combination of these factors [4]. Considerable overlap exists between these processes, especially sarcopenia and cachexia in frail older adults with comorbidities and chronic disease [5]; however, in more robust individuals where muscle and disease-related processes may be less relevant, the primary forward indicators of malnutrition risk in this group are likely to center on determinants such as anorexia and inadequate dietary intake.

Over the last few decades, multiple screening tools have been developed to assess nutritional status, for example Mini-Nutritional Assessment (MNA), Malnutrition Universal Screening Tool (MUST), Nutritional Risk Screening 2002 (NRS-2002), and Geriatric Nutritional Risk Index (GNRI). While these tools are comprehensive and have been validated in various settings, there remains a need to strike a balance between efficacy and efficiency, especially for resource-intensive tools that require trained assessors or laboratory measurements [6,7,8]. These tools also do not specifically evaluate anorexia, which has been associated with increased risk for weight loss, malnutrition, and reduced quality of life in older adults [9]. A robust screening tool to evaluate appetite could serve as an early marker of malnutrition risk and downstream consequences of frailty and functional decline in robust older adults, and hence create opportunities for early intervention.

The Simplified Nutritional Appetite Questionnaire (SNAQ) and the Council on Nutrition Appetite Questionnaire (CNAQ) are self-administered questionnaires adapted from the Appetite, Hunger and Sensory Perception questionnaire (AHSP), an appetite assessment tool validated among community-dwelling older adults in the Netherlands [10]. The shorter four-item SNAQ comprising items 1, 2, 4 and 6 of the CNAQ was shown to have good reliability, sensitivity and specificity to predict malnutrition in both specialized and nonspecialized older adult populations [11,12,13,14,15,16,17,18,19,20,21,22,23,24,25]. Prior utilization of the SNAQ, cutoffs and factor analysis results have been summarized in Table 1.

However, gaps remain in our understanding of the diagnostic performance and optimal cutoff of SNAQ in robust older adults in the community where the prevalence of malnutrition is lower. The good diagnostic performance of SNAQ and the recommended cutoff of ≤14 was derived from community studies which were primarily based on older adults who were either frail with comorbidities [18,20,22] or who were younger in age [17,23]. There also exists uncertainty in the psychometric properties of SNAQ, such that its factor structure and validity in more robust populations cannot be assumed. Earlier studies highlighted that the item on food intake correlates poorly with total SNAQ score [21], and that reliability of SNAQ increases if this item was omitted [15]. In addition, the one-factor solution of SNAQ was derived either from studies in older adults with health-seeking behavior [14] or specialized populations [17,21,23].

The aim of this study is thus to determine an optimal cutoff for SNAQ in screening for malnutrition in healthy community-dwelling older adults. This study also seeks to examine the factor structure of SNAQ, as well as assess its psychometric properties including validity and reliability.

## 2. Materials and Methods

### 2.1. Study Population

The “Longitudinal Assessment of Biomarkers for characterization of early Sarcopenia and Osteosarcopenic Obesity in predicting frailty and functional decline in community-dwelling Asian older adults Study” (GeriLABS 2) is a prospective cohort study involving cognitively intact and functionally independent community-dwelling adults aged 50 years and older in Singapore. We recruited 230 participants from December 2017 to March 2019. Inclusion criteria were as follows: (i) aged 50 to 99 years at study enrolment, (ii) community-dwelling, (iii) independent in both basic and instrumental activities of daily living (ADLs), and (iv) generally healthy as defined by a score of <3 on the FRAIL criteria. The FRAIL scale comprises 5 components: Fatigue, Resistance, Ambulation, Illnesses & Loss of Weight with a total score of 0–5 points and represents frail (3–5), pre-frail (1–2) and robust (0) health status [26]. Participants were excluded if they had cognitive impairment (prior diagnosis of dementia or modified Chinese version of Mini-Mental State Examination (CMMSE) score ≤21) [27], unable to walk 8 m independently, or were living in a long-term residential care facility. This study reports cross-sectional data from the point of recruitment into the study.

### 2.2. Clinical Assessment

We collected demographic data and information on cardiovascular and bone health. Anthropometric measurements (standing height and body weight to calculate Body Mass Index (BMI), calf circumference, mid-arm circumference and waist circumference) were collected. Cognition was assessed via the CMMSE [27]. Mood was assessed via the Geriatric Depression Scale (GDS), with a locally-validated cutoff score of ≥4 to distinguish presence of depressive symptoms [28]. Functional status was assessed using the Barthel ADL index [29] and Lawton and Brody’s instrumental ADL index [30], while activity level was evaluated via the Frenchay Activities Index (FAI) [31] and International Physical Activity Questionnaire (IPAQ) [32]. We also measured life-space mobility using the Life-Space Assessment (LSA), which comprises five space-levels corresponding to activities outside the bedroom, home, neighborhood, town, and beyond, respectively [33]. Using cohort quintile cutoffs, we defined low physical activity as FAI ≤29 and IPAQ <2826 METS respectively, and low life-space mobility as LSA <76 [34].

Physical frailty was assessed via the modified Fried phenotypic criteria, with a score of 0 denoting nonfrail, 1–2 denoting prefrail, and 3 and above denoting physical frailty [35]. Details of the operationalization of the modified Fried phenotypic criteria have been previously described [36]. Social frailty was assessed via the locally-validated eight-item Social Frailty Scale (SFS-8), which measures the three domains of social resources, social activities and financial resources, and social need fulfilment (score range: 0–8 points). A score of 0–1 denotes social nonfrailty, 2–3 denotes social prefrailty, and a score of 4 and above denotes social frailty [34].

Physical function was assessed via the Short Physical Performance Battery (SPPB) [37], maximal hand grip strength using a hydraulic hand dynamometer, usual gait speed on the three-meter walk test, and the five-time chair stand test. The SPPB score of <11 denoted poorer quality of life and also corresponded to the quintile cutoff in our cohort [38]. Cutoffs for maximal hand grip strength (<28 kg for males, <18 kg for females), gait speed (<1.0 m/s) and five-time chair stand test (≥12 s) were based upon The Asian Working Group for Sarcopenia (AWGS) 2019 guidelines [39].

### 2.3. Nutritional Assessment

Nutritional risk was assessed via the Simplified Nutritional Appetite Questionnaire (SNAQ), a four-item tool comprising items 1, 2, 4 and 6 of the CNAQ [11]. These items assess appetite, satiety, taste of food and number of meals per day respectively. The SNAQ was developed as a self-assessment screening tool that is quick and easy to administer without the need for trained assessors or laboratory measurements. The total score ranges from 4 to 20. Prior validation studies suggest a cutoff of ≤14 to predict malnutrition and involuntary weight loss (Table 1).

We compared SNAQ against the Mini Nutritional Assessment (MNA), which comprises both screening and nutritional assessment items. The MNA has been validated in various settings with high reliability, sensitivity and specificity [40,41]. A cutoff score of <24 on the MNA-long form indicates risk of malnutrition [42]. Laboratory markers of 25-hydroxy Vitamin D and serum albumin levels were also collected.

### 2.4. Statistical Analysis

We performed statistical analyses using IBM SPSS Statistics version 23.0 (IBM Corporation, Armonk, NY, USA). All statistical tests were two-tailed, with *p* < 0.05 considered statistically significant. Continuous variables were expressed as means (standard error) or as medians (interquartile range). Categorical variables were expressed as counts and percentages.

To derive the optimal cutoff for SNAQ, we performed receiver operating characteristic curve (ROC) analysis against MNA-long form <24 as the reference standard. We calculated the optimal point between sensitivity and specificity using Youden index. Diagnostic performance was ascertained via area under ROC curve.

Internal consistency of the scale was assessed using Cronbach’s alpha. To ascertain the factor structure of SNAQ, we conducted exploratory factor analysis (EFA) using the Kaiser-Meyer-Olkin (KMO) statistic as a measure of sampling adequacy and the Bartlett’s test of sphericity to ascertain necessity to perform a factor analysis. We conducted principal component analysis with varimax rotation to ascertain the underlying factor structure. The number of factors to be retained was determined by parallel analysis, which was less likely to over-estimate the number of factors [43]. Items with factorial loadings <0.4 were eliminated.

To assess the construct validity of SNAQ, we analyzed convergent (i.e., MNA, calf circumference, GDS) and discriminant (i.e., waist circumference, CMMSE, level of education) validity for SNAQ total and factor scores via correlational analysis using Spearman’s rho. For concurrent validity, we ascertained known-groups validity by examining differences in mean SNAQ scores against depressive symptoms (GDS <4 vs. ≥4) via independent samples t-test, as well as social frailty (nonfrail, prefrail and frail) and physical frailty (nonfrail, prefrail and frail) via one-way analysis of variance with Bonferroni correction for post-hoc comparisons. Predictive validity of SNAQ was assessed via logistic regression, with adjustments for age, gender, education and MNA score, for the outcomes of life-space mobility, social frailty, SPPB, handgrip strength and five-time chair stand. We compared the suggested SNAQ cutoff of ≤14 against our derived cutoff of ≤15 in this study.

### 2.5. Ethics Approval and Consent

The study was conducted in accordance with the Declaration of Helsinki, and the protocol was approved by the Domain Specific Review Board of the National Healthcare Group (DSRB Ref: 2017/00850). Written consent was obtained from all participants prior to study participation.

## 3. Results

### 3.1. Baseline Characteristics

We studied 230 participants with mean age 67.2 ± 7.4 years and mean education of 10.8 ± 4.4 years (Table 2). A total of 72.6% were female and 92.2% were of Chinese ethnicity. The high cognitive scores (CMMSE, mean ± SD: 26.1 ± 1.7), ADL indices (basic and instructional ADLs, mean score 100 and 23 respectively) and activity levels (FAI mean ± SD: 32.2 ± 5.2 and IPAQ 5023.4 ± 2402.7 metabolic equivalents (METS) per week) attested to the relatively robust health of participants. Based on the modified Fried frailty phenotype, only two (0.9%) participants were classified as physically frail, with the majority (41.3% and 57.8% respectively) classified as prefrail and robust. Based on the SFS-8, 17 (7.4%) participants were classified as socially frail, with 28.8% socially prefrail and 63.8% socially nonfrail. The median GDS score was 1 (interquartile range, IQR = 0–2).

The prevalence of risk of malnutrition in this study was 5.7% based on the MNA cutoff of <24. The risk of malnutrition identified by SNAQ based on cutoff scores of ≤14 and ≤15 was 18.3% and 40.4% respectively. In comparing participants identified by SNAQ to be at risk of malnutrition (i.e., SNAQ total score ≤15, *n* = 93) against those who were not at risk (*n* = 137), the differences in age, calf circumference, GDS, MNA, life space (levels 3, 5 and total score), social frailty, SPPB, hand grip strength, repeated chair stand test were significant (all *p* < 0.05).

### 3.2. SNAQ Cutoff Score

**Figure 1:**
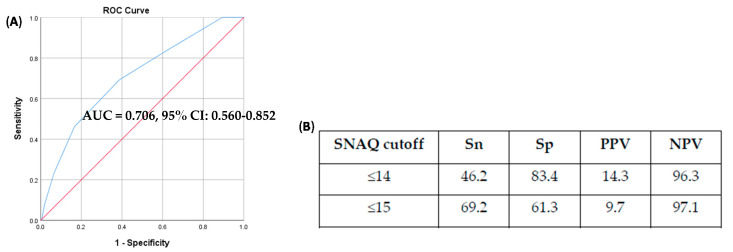
Receiver operating characteristic (ROC) curve (**A**) and derivation of simplified nutritional appetite questionnaire (SNAQ) cutoff score (**B**). AUC: Area under ROC curve; CI: Confidence Interval; Sn: Sensitivity; Sp: Specificity; PPV: Positive Predictive Value; NPV: Negative Predictive Value. The blue line is the ROC curve; the red line depicts the line where AUC = 0.5.

The area under ROC curve was 0.706 in our study (Figure 1). The optimal cutoff based on the Youden index was SNAQ ≤15, with a corresponding sensitivity of 69.2% and specificity of 61.3% (positive predictive value, PPV = 9.7%; negative predictive value; NPV = 97.1%). In contrast, using the cutoff of ≤14, the sensitivity and specificity were 46.2% and 83.4% respectively (PPV = 14.3%; NPV = 96.3%). 

### 3.3. Factor Structure and Reliability of SNAQ

Factor analysis was appropriate as the KMO statistic was 0.530, and the Bartlett’s test of sphericity was 32.553 (*p* < 0.001). The optimal number of factors recommended by parallel analysis was two. The two-factor structure of the SNAQ accounted for 61.5% of the total variance. Factor 1 comprised two items representing appetite perception and accounted for 34.9% of variance. Factor 2 comprised two items representing satiety and intake, and accounted for 26.6% of variance (Table 3). The Cronbach’s alpha of SNAQ was 0.333. There was a slight increase in Cronbach’s alpha if item 4 was deleted.

### 3.4. Convergent and Discriminant Validity

SNAQ Factors 1 and 2 correlated strongly with the total SNAQ score (r = 0.767 and 0.689 respectively, *p* < 0.05). The correlation between Factors 1 and 2 was poor (r = 0.119, *p* > 0.05) (Table 4). In terms of convergent and discriminant validity, the strength of correlations was low to moderate in this sample of healthy older adults. SNAQ total and factor scores correlated with MNA, calf circumference and GDS, with stronger correlations for SNAQ total and Factor 1 scores (range of r: 0.151 to 0.238, *p* < 0.05) compared to Factor 2 (range of r: 0.049 to 0.161, *p* <0.05 only for correlation with MNA). The correlations for waist circumference, CMMSE and education were weak (range of r: 0.005 to 0.147, *p* > 0.05 except for Factor 1 correlation with CMMSE), thus corroborating the discriminant validity of SNAQ.

### 3.5. Concurrent Validity

SNAQ total score showed a decreasing trend with increasing social frailty (15.96 vs. 15.56 vs. 15.18, *p* = 0.045), physical frailty (15.94 vs. 15.61 vs. 14.50, *p* > 0.05) and depressive symptoms (15.84 vs. 15.07, *p* > 0.05) (Table 5). This attests to the concurrent validity of the SNAQ. For the factor scores, there was a similar trend observed for Factor 1, with a significant decreasing trend in Factor 1 scores with increasing social frailty (7.91 vs. 7.70 vs. 6.94, *p* = 0.001). A similar trend was observed for Factor 2 with physical frailty and depressive symptoms (*p* > 0.05), but not for social frailty (8.05 vs. 7.86 vs. 8.24, *p* > 0.05).

### 3.6. Predictive Validity and Outcome Associations

In logistic regression analysis adjusted for age, gender, education and MNA, both SNAQ cutoffs of ≤14 and ≤15 were associated with life-space mobility (odds ratio, OR 2.29 vs. 2.06, *p* = 0.041 for both), SPPB (OR 3.93, *p* = 0.003 vs. OR 3.00, *p* = 0.010) and five-time chair stand test (OR 2.45, *p* = 0.029 vs. OR 2.18, *p* = 0.028) (Table 6). In contrast, only the ≤15 cutoff was significantly associated with social frailty (OR 1.99, *p* = 0.025 vs. OR 1.05, *p* = 0.890 for SNAQ ≤14). For the outcome of hand grip strength, the ≤15 cutoff had higher odds ratios albeit not statistically significant.

## 4. Discussion

To our knowledge, this is the first study to ascertain the optimal cutoff, factor structure and validity of SNAQ in healthy, cognitively-intact and functionally-independent community-dwelling older adults. Our findings suggest an optimal SNAQ cutoff of ≤15 to screen for risk of malnutrition that is higher than the currently recommended ≤14. In healthy older persons, the higher SNAQ cutoff lowers the threshold for detection of anorexia, thus improving its diagnostic performance as a screening tool by increasing sensitivity to rule out false negative cases of anorexia for further evaluation. In one study involving hospitalized older adults and their spouses, raising the SNAQ cutoff from ≤14 to ≤15 similarly increased sensitivity from 70.8% to 79.2%, albeit also at the expense of specificity [13]. A SNAQ cutoff of ≤15 could thus present an opportunity for early case detection of malnutrition risk in relatively healthy individuals, and consequently early nutrition assessment, education and intervention to prevent adverse health outcomes and allow robust older adults to remain independent in the community [44,45,46].

Our study also found a two-factor structure of the SNAQ, which differs from prior validation studies that suggest a unifactorial model of ‘Appetite’ [14,17,21,23]. The factors identified in our study (i.e., Factor 1: Appetite Perception; Factor 2: Satiety and Intake) accounted for a higher proportion of the total variance (61.5%) compared with earlier studies (33.7% to 54.0%), and corresponded to the premise that the primary forward indicators of malnutrition risk in healthy older adults are likely to be driven more by anorexia and inadequate dietary intake, than by muscle or disease-related processes such as sarcopenia and cachexia [4]. Between the two factors, convergent and concurrent validity of the SNAQ appear to be driven more by Factor 1 than Factor 2, which is in keeping with prior studies that have identified ‘Appetite’ as the main outcome measure in the SNAQ. This suggests that while a distinct factor on its own, satiety and intake (i.e., Factor 2) may be less discriminatory in a relatively robust population, and may also be subjected to differences in culture, socioeconomic status or varying perceptions of meal quantity and frequency that are considered to be norms [47,48,49]. The contribution of Factor 2 may also explain the relatively-low reliability of SNAQ in our study (Cronbach’s alpha 0.333); in healthy older adults, the brief 4-item SNAQ may not be sufficiently reliable in screening for the risk of malnutrition, and there may be a need to consider supplementing with other items that examine determinants such as the social facilitation of eating and economic determinants of nutrition [50,51,52].

In line with this, we found that SNAQ was significantly associated with social frailty, life-space mobility and muscle function. Notably, a higher SNAQ cutoff of ≤15 showed a stronger association with social frailty compared to the traditional cutoff of ≤14. These observations remained significant even after adjustment for MNA, suggesting that anorexia (as measured by SNAQ) predicts risk of malnutrition above and beyond the effect of MNA in healthy older adults. This finding also reinforces the relationship between appetite and social influences, and echoes increasing evidence that social eating norms play a role in the development of healthy eating behavior and maintenance of adequate nutrition, especially in later life [53,54]. While many factors contribute to poor appetite (e.g., disease, drugs, depression, socioeconomic factors, impaired masticatory function) [55], in robust older adults without comorbid diseases, the predominant precursor of malnutrition risk may be related to the concept of anorexia of aging instead [56]. Anorexia of the aging involves physiological processes such as age-related neurohormonal changes (e.g., decline in central orexigenic neuropeptide activity, increase in cholecystokinin levels), decline in taste and smell, and reduced antral stretch that collectively lead to a notable decline in food intake with age, and represents an entity that exists discrete from pathological causes of anorexia [9,57,58,59]. Anorexia can lead to malnutrition and weight loss, which in turn increases the risk of frailty, disability and mortality [60,61,62]. This highlights the importance of screening for anorexia as a forward indicator of risk of malnutrition, even in healthy older adults; a good example is the incorporation of SNAQ as part of the Rapid Geriatric Assessment to screen for malnutrition or anorexia as a geriatric syndrome alongside physical frailty, sarcopenia and cognitive impairment [63].

Some limitations in this study are worth highlighting. First, in terms of the predictive validity of SNAQ, the cross-sectional analysis limits definitive conclusions about causality, and reverse causality to account for the observed associations cannot be excluded. Next, our study comprised predominantly Chinese participants who were cognitively-intact, independent and nonfrail. These findings may not be generalizable to other non-Chinese Asian settings with more frail older adults, or communities with culturally-diverse eating behaviors which may limit interpretation of Factor 2 (i.e., satiety and intake) in the two-factor SNAQ structure. Finally, the low prevalence of risk of malnutrition (5.7%) in our study, which is consistent with the more robust health status of study participants, may have decreased the reliability of SNAQ compared to prior validation studies. We recommend more studies in different populations to examine the influence of socio-cultural characteristics on factor structure and reliability of SNAQ as a screening tool for risk of malnutrition in robust older adults.

## 5. Conclusions

In conclusion, our study found a higher optimal cutoff of ≤15 for SNAQ as a screening tool for risk of malnutrition in healthy community-dwelling older adults. Compared with the recommended cutoff of ≤14, the higher cutoff improves diagnostic performance by increasing sensitivity of SNAQ for anorexia detection to facilitate earlier detection of malnutrition risk in healthy older adults for timely nutritional assessment and intervention. Our study adds to the growing body of evidence regarding the psychometric properties of SNAQ, by explicating a two-factor structure comprising ‘Appetite Perception’ and ‘Satiety and Intake’ and affirming the validity of SNAQ in healthy older adults. Importantly, we also demonstrated the association of anorexia with other important health outcomes such as social frailty, life-space mobility and muscle function. Our findings set the stage for further longitudinal studies among relatively robust community-dwelling older adults to corroborate the predictive validity for adverse outcomes associated with malnutrition risk, and to further delineate the sociocultural and pathophysiological mechanisms that underpin the relationship between anorexia and risk of malnutrition.

## Figures and Tables

**Table 1:** Summary of Simplified Nutritional Appetite Questionnaire (SNAQ) Studies [11,12,13,14,15,16,17,18,19,20,21,22,23,24,25].

Reference	Setting	Study Population	Comparator	SNAQ Cutoff	AUC	Sn (%)	Sp (%)	α	Comments
**Non-Specialised Study Populations**									
Wilson 2005 [11]	Community-dwelling subjects, USA	*N* = 352 Mean age = 73.8 Malnutrition = 10.5%	AHSP	<14	0.870	81.6	84.6	0.740	Analysis was done on the older community-dwelling group. In younger community-dwelling subjects (mean age 39.4 ± 12.0 years), the optimal SNAQ cutoff was <15 (Sn 79.2%, Sp 79.4%)
Hanisah 2012 [12]	Subjects from the Medical Ward and Outpatient Medical Clinic in a hospital, Malaysia	*N* = 145 Mean age = 68.3 Malnutrition = 61.0%	AHSP	≤14		69.7	62.5	0.578	
Rolland 2012 [13]	Hospitalized older patients in geriatric units and their spouses, France	*N* = 175 Mean age = 77.8 Malnutrition = 27.4%	MNA-LF	≤14 ≤15	0.767	70.8 79.2	74.4 57.5		
Nakatsu 2015 [14]	Community-dwelling subjects recruited for a health check, Japan	*N* = 84 Mean age = 76.4 Malnutrition = N/A	MNA-SF	<14				0.545	Translated into Japanese. EFA showed 1 factor (50.0% variance)
Ilhan 2018 [15]	Community-dwelling subjects attending a Geriatrics outpatient clinic, Turkey	*N* = 442 Mean age = 77.1 Malnutrition = 28.7%	MNA-LF	≤14				0.522	Translated into Turkish. Cronbach’s α is increased (0.757) if SNAQ Q4 is deleted.
Akin 2019 [16]	Community-dwelling subjects, Turkey	*N* = 871 Mean age = 71.4 Malnutrition = 45.5%	MNA-LF	≤14	0.725	50.0	84.0	0.639	
Lau 2020	Community-dwelling subjects, Singapore	*N* = 230 Mean age = 67.2 Malnutrition = 5.7%	MNA-LF	≤15	0.706	69.2	38.7	0.333	EFA showed 2 factors (61.5% variance).
**Specialised study populations**									
Sties 2012 [17]	Subjects of a metabolic and cardiopulmonary rehabilitation programme, Brazil	*N* = 145 Mean age = 63.0 (males), 66.0 (females) Malnutrition = 7.0%						0.610	Translated into Portuguese. EFA showed 1 factor (47.0% variance). SNAQ Q4 correlated poorly with SNAQ total score.
Andreae 2015 [18]	Subjects with heart failure attending an outpatient heart failure clinic, Sweden	*N* = 186 Median age = 72.0 Malnutrition = N/A						0.770	Translated into Swedish. CFA (single-factor model): Chi-square 3.0, *p* = 0.368; RMSEA 0.05 (90% CI 0.00-0.16), *p* = 0.368; CFI 1.00, TLI 0.99, WRMR 0.30.
Yaxley 2015 [19]	Subjects receiving ambulatory rehabilitation following an acute hospital admission, Australia	*N* = 185 Median age = 78.0 Malnutrition = 63%	MNA-LF	≤14		28.0	94.0		
Helfenstein 2016 [20]	Subjects with metastatic cancer receiving outpatient palliative care, Switzerland	*N* = 118 Median age = 69.0 Malnutrition = 31%	NRS	≤14	0.653	38.0	66.0		Translated into German.
Tokudome 2017 [21]	Community-dwelling subjects attending day care or health promotion classes, on meal delivery services; and subjects staying in group homes, Japan	*N* = 649 Mean age = 80.4 Malnutrition = N/A	MNA-SF	≤14				0.640	Translated into Japanese. EFA showed 1 factor (33.7% variance). SNAQ Q4 correlated poorly with SNAQ total score.
Wang 2018 [22]	Hospitalized subjects with liver cirrhosis, China	*N* = 70 Mean age = 66.7 Malnutrition = 75.7%	BMI	≤11.5	0.702	79.2	72.7		
Mohammadi 2019 [23]	Subjects seeking weight reduction at a private nutrition clinic, Iran	*N* = 213 (all females) Mean age = 39.0 Malnutrition = N/A						0.700	Translated into Farsi. EFA showed 1 factor (54.0% variance).
Oh 2019 [24]	Subjects with recurrent or metastatic cancer, Korea	*N* = 194 Mean age = 60.0 Malnutrition = 31.4%	MNA-SF	≤14		56.5	44.3	0.737	Translated into Korean. EFA showed no overlapping concept item among SNAQ Q1-4.
Wleklik 2019 [25]	Hospitalized subjects with heart failure, Poland	*N* = 103 Median age = 65.0–70.0 Malnutrition = 94%	MNA-LF	≤14				0.860	Translated into Polish. CFA (single-factor model): Chi-square 1.93, *p* = 0.380; RMSEA 0.00 (90% CI: 0.00-0.19), *p* = 0.47; CFI 1.00, TLI 1.00, SRMR 0.02.

AHSP: Appetite, Hunger and Sensory Perception Questionnaire; AUC: Area under ROC curve; BMI: Body mass index; CFA: Confirmatory factor analysis; CFI: Comparative Fit Index; CI: Confidence Interval; EFA: Exploratory factor analysis; MNA-LF: Mini-Nutritional Assessment Long Form; MNA-SF: Mini-Nutritional Assessment Short Form; NRS: Nutrition Risk Score; RMSEA: Root Mean Square Error of Approximation; SRMR: Standardized Root Mean Square Residual; Sn: Sensitivity; Sp: Specificity; TLI: Tucker Lewis Index; WRMR: Weighted Root Mean Square Residual, N/A: The rate of malnutrition was not specified in these studies.

**Table 2:** Characteristics of Study Cohort.

Characteristics of Study Cohort	Total (*n* = 230)	SNAQ >15 (*n* = 137)	SNAQ ≤15 (*n* = 93)	*p*-Value
Age (years)	67.2 ± 7.4	67.0 ± 6.8	67.5 ± 8.3	0.032
Gender (female, %)	72.6	69.3	77.4	0.178
Race (Chinese, %)	92.2	93.4	90.3	0.426
Education (years)	10.8 ± 4.4	11.0 ± 4.6	10.4 ± 4.0	0.079
No. of cardiovascular risk factors (%) *				0.696
None	29.7	28.7	31.2	
1 to 2	55.9	58.0	52.7	
3 and above	14.4	13.3	16.1	
Known osteoporosis (%)	27.4	27.0	28.0	0.874
Anthropometry				
BMI (kg/m^2^)	23.9 ± 3.2	23.9 ± 3.2	23.8 ± 3.3	0.625
Calf circumference (cm)	34.8 ± 3.2	35.1 ± 3.1	34.4 ± 3.3	0.729
Calf circumference < 31 cm (%)	4.3	1.5	8.6	0.009
Mid-arm circumference (cm)	27.6 ± 3.0	27.8 ± 2.9	27.4 ± 3.1	0.936
Waist circumference (cm)	85.3 ± 9.3	85.4 ± 9.0	85.0 ± 9.8	0.426
Cognition and Mood				
CMMSE (0–28)	26.1 ± 1.7	26.3 ± 1.6	25.9 ± 1.8	0.311
GDS (0–15)	1.0 (0.0–2.0)	1.0 (0.0–1.0)	1.0 (0.0–2.0)	0.002
Nutrition				
MNA total (0–30)	27.2 ± 1.9	27.6 ± 1.6	26.6 ± 2.1	0.011
MNA < 24: Malnourished or at risk of malnutrition (%)	5.7	2.9	9.7	0.029
SNAQ total (0–20)	15.8 ± 1.5	16.8 ± 0.8	14.3 ± 0.9	0.110
SNAQ ≤ 14 (%)	18.3	-	-	
SNAQ ≤ 15 (%)	40.4	-	-	
Functional Status and Activity Level				
Barthel ADL index (0–100)	100.0 (95.0–100.0)	100.0 (95.0–100.0)	100.0 (95.0–100.0)	0.094
Lawton iADL scale (0–23)	23.0 (22.0–23.0)	23.0 (22.0–23.0)	23.0 (22.0–23.0)	0.449
Frenchay Activities Index, FAI (0–45)	32.2 ± 5.2	32.8 ± 4.8	31.3 ± 5.6	0.302
IPAQ, total METS per week (mins)	5042.4 ± 2402.7	5429.0 ± 2506.9	4477.2 ± 2131.0	0.086
Life-Space Assessment				
Life-Space Level 1 (max 8)	8.0 ± 0.3	8.0 ± 0.17	8.0 ± 0.41	0.474
Life-Space Level 2 (max 16)	15.4 ± 2.2	15.6 ± 1.6	15.0 ± 2.7	0.069
Life-Space Level 3 (max 24)	18.7 ± 6.9	19.5 ± 6.7	17.6 ± 7.1	0.041
Life-Space Level 4 (max 32)	21.5 ± 8.5	22.0 ± 8.8	20.8 ± 8.0	0.285
Life-Space Level 5 (max 40)	26.9 ± 9.7	28.3 ± 9.4	24.7 ± 9.7	0.004
Life-Space Total (max 120)	90.5 ± 17.7	93.4 ± 16.5	86.1 ± 18.6	0.002
Frailty and Physical Function				
Modified Fried phenotypic criteria (0–5)				0.289
0: Nonfrail (%)	57.8	62.0	51.6	
1–2: Prefrail (%)	41.3	37.2	47.3	
3 and above: Frail (%)	0.9	0.7	1.1	
Social Frailty, SFS-8 (0–8)				0.004
0–1: Social nonfrailty (%)	63.8	72.1	51.6	
2–3: Social prefrailty (%)	28.8	23.5	36.6	
4 and above: Social frailty (%)	7.4	4.4	11.8	
SPPB (0–12)	12.0 (11.0–12.0)	12.0 (12.0–12.0)	12.0 (11.0–12.0)	0.001
Hand grip strength (kg)	23.5 ± 7.1	24.5 ± 7.7	21.9 ± 5.9	0.017
Gait speed (m/s)	1.2 ± 0.2	1.2 ± 0.2	1.1 ± 0.2	0.256
5-time chair stand test (secs)	9.46 ± 3.03	9.05 ± 2.81	10.06 ± 3.25	0.014
Laboratory Markers				
25-hydroxy Vitamin D level (µg/L)	30.6 ± 8.8	30.6 ± 8.4	30.6 ± 9.4	0.322
Serum albumin (g/L)	41.2 ± 2.6	41.3 ± 2.6	41.0 ± 2.6	0.890

Mean ± SD for variables with normal distribution; median (interquartile range) for non-normal variables. * Cardiovascular risk factors: Hypertension, hyperlipidemia, diabetes mellitus, ischemic heart disease/congestive cardiac failure/myocardial infarction, atrial fibrillation, stroke or transient ischemic attack, peripheral vascular disease, smoking. ADL: Activities of Daily Living; iADL: Instrumental Activities of Daily Living; BMI: Body Mass Index; CMMSE: Chinese Mini-Mental Status Examination; GDS: Geriatric Depression Scale; IPAQ: International Physical Activity Questionnaire; MNA: Mini Nutritional Assessment; SNAQ: Simplified Nutritional Appetite Questionnaire; SFS-8: Social Frailty Scale 8-item; SPPB: Short Physical Performance Battery.

**Table 3:** Factor Structure and Internal Consistency of SNAQ.

SNAQ Items	Mean ± SD (*n* = 230)	Factor 1: Appetite Perception	Factor 2: Satiety and Intake	α If Item Deleted
Q1: My appetite is ____	3.97 ± 0.71	0.811		0.214
Q2: When I eat, I feel full ____	3.80 ± 0.57		0.676	0.285
Q3. Food tastes ____	3.81 ± 0.60	0.797		0.179
Q4. Normally I eat ____	4.21 ± 0.72		0.808	0.398 *
Eigenvalue		1.396	1.063	
Explained variance (%)		34.9	26.6	

Factorial loading values of <0.4 are not displayed in the table. * Cronbach’s α if item deleted is increased compared with original scale.

**Table 4:** Convergent and Discriminant Validity of SNAQ.

	SNAQ Total	SNAQ Factor 1	SNAQ Factor 2
SNAQ Factor 1	0.767 *		
SNAQ Factor 2	0.689 *	0.119	
Convergent			
MNA	0.238 *	0.187 *	0.161 *
Calf circumference	0.157 *	0.151 *	0.049
GDS	−0.241 *	−0.257 *	−0.074
Discriminant			
Waist circumference	0.037	0.011	0.047
CMMSE	0.127	0.147 *	0.052
Education	−0.089	0.128	0.005

* *p*-Value < 0.05. CMMSE: Chinese Mini-Mental Status Examination; GDS: Geriatric Depression Scale; MNA: Mini Nutritional Assessment; SNAQ: Simplified Nutritional Appetite Questionnaire.

**Table 5:** Concurrent Validity of SNAQ.

	Social Frailty (SFS-8)				Physical Frailty (Fried)				Depressive Symptoms (GDS)		
	Social Nonfrailty (*n* = 146)	Social Prefrailty (*n* = 66)	Social Frailty (*n* = 17)	*p*	Nonfrail (*n* = 133)	Prefrail (*n* = 95)	Frail (*n* = 2)	*p*	No (*n* = 222)	Yes (*n* = 8)	*p*
SNAQ Total	15.96 ± 1.35	15.56 ± 1.78	15.18 ± 1.43	0.045	15.94 ± 1.39	15.61 ± 1.64	14.50 ± 2.12	0.127	15.84 ± 1.50	15.07 ± 1.54	0.065
SNAQ Factor 1	7.91 ± 0.95	7.70 ± 1.18	6.94 ± 1.09	0.001	7.91 ± 1.01	7.63 ± 1.12	7.00 ± 1.41	0.087	7.81 ± 1.05	7.43 ± 1.22	0.195
SNAQ Factor 2	8.05 ± 0.89	7.86 ± 1.12	8.24 ± 0.97	0.267	8.03 ± 0.90	7.98 ± 1.06	7.50 ± 0.71	0.706	8.03 ± 0.07	7.64 ± 0.27	0.150

GDS: Geriatric Depression Scale; Fried: Modified Fried phenotypic criteria; SFS-8: Social Frailty Scale 8-item.

**Table 6:** Logistic Regression Models for Outcomes: Comparison between SNAQ ≤ 14 vs. SNAQ ≤ 15.

	Unadjusted		Adjusted *	
Outcome Variables	Odds Ratio (95% CI)	*p*	Odds Ratio (95% CI)	*p*
Social frailty, SFS-8 ≥ 2				
SNAQ ≤14	1.59 (0.81, 3.13)	0.182	1.05 (0.50, 2.24)	0.890
SNAQ ≤15	2.42 (1.39, 4.20)	0.002	1.99 (1.09, 3.63)	0.025
Life space mobility, LSA total ≤76				
SNAQ ≤14	3.12 (1.50, 6.48)	0.002	2.29 (1.03, 5.07)	0.041
SNAQ ≤15	2.68 (1.39, 5.16)	0.003	2.06 (1.03, 4.12)	0.041
Short Physical Performance Battery <11				
SNAQ ≤14	3.78 (1.65, 8.64)	0.002	3.93 (1.60, 9.66)	0.003
SNAQ ≤15	2.94 (1.33, 6.52)	0.008	3.00 (1.30, 6.94)	0.010
Hand grip strength ^				
SNAQ ≤14	1.39 (0.66, 2.95)	0.390	0.79 (0.33, 1.87)	0.587
SNAQ ≤15	1.51 (0.82, 2.79)	0.188	1.14 (0.58, 2.25)	0.711
Five-time chair stand test ≥12 s				
SNAQ ≤14	2.53 (1.20, 5.35)	0.015	2.45 (1.10, 5.48)	0.029
SNAQ ≤15	2.15 (1.11, 4.16)	0.023	2.18 (1.09, 4.38)	0.028

* Model adjusted for age, gender, education and Mini-Nutritional Assessment (MNA). ^ Asian Working Group for Sarcopenia (AWGS) 2019 definition: Maximal hand grip strength <28 kg (males) and <18 kg (females). LSA: Life Space Assessment; SFS-8: Social Frailty Scale 8-item, SPPB: Short Physical Performance Battery.

## References

[1] Wallace J.I., Schwartz R.S., LaCroix A.Z., Uhlmann R.F., Pearlman R.A. (1995). Involuntary Weight Loss in Older Outpatients: Incidence and Clinical Significance. J. Am. Geriatr. Soc..

[2] Roberts H.C., Lim S.E.R., Cox N.J., Ibrahim K. (2019). The Challenge of Managing Undernutrition in Older People with Frailty. Nutrients.

[3] Norazman C.W., Adznam S.N., Jamaluddin R. (2020). Malnutrition as Key Predictor of Physical Frailty among Malaysian Older Adults. Nutrients.

[4] Hickson M. (2006). Malnutrition and Ageing. Postgrad. Med. J..

[5] Gingrich A., Volkert D., Kiesswetter E., Thomanek M., Bach S., Sieber C.C., Zopf Y. (2019). Prevalence and Overlap of Sarcopenia, Frailty, Cachexia and Malnutrition in Older Medical Inpatients. BMC Geriatr..

[6] Guaitoli P.R., Jansma E.P., de Vet H.C. (2014). Nutrition Screening Tools: Does One Size Fit All? A Systematic Review of Screening Tools for the Hospital Setting. Clin. Nutr..

[7] Power L., Mullally D., Gibney E.R., Clarke M., Visser M., Volkert D., Bardon L., de van der Schueren M.A.E., Corish C.A. (2018). A Review of the Validity of Malnutrition Screening Tools Used in Older Adults in Community and Healthcare Settings—A MaNuEL Study. Clin. Nutr. ESPEN.

[8] Dent E., Hoogendijk E.O., Visvanathan R., Wright O.R.L. (2019). Malnutrition Screening and Assessment in Hospitalised Older People: A Review. J. Nutr. Health Aging.

[9] Morley J.E. (2012). Anorexia of Aging: A True Geriatric Syndrome. J. Nutr. Health Aging.

[10] Mathey M.F. (2001). Assessing Appetite in Dutch Elderly with the Appetite, Hunger and Sensory Perception (AHSP) Questionnaire. J. Nutr. Health Aging.

[11] Wilson M.-M.G., Thomas D.R., Rubenstein L.Z., Chibnall J.T., Anderson S., Baxi A., Diebold M.R., Morley J.E. (2005). Appetite Assessment: Simple Appetite Questionnaire Predicts Weight Loss in Community-Dwelling Adults and Nursing Home Residents. Am. J. Clin. Nutr..

[12] Hanisah R., Shahar S., Lee F.S. (2012). Validation of Screening Tools to Assess Appetite among Geriatric Patients. J. Nutr. Health Aging.

[13] Rolland Y., Perrin A., Gardette V., Filhol N., Vellas B. (2012). Screening Older People at Risk of Malnutrition or Malnourished Using the Simplified Nutritional Appetite Questionnaire (SNAQ): A Comparison With the Mini-Nutritional Assessment (MNA) Tool. J. Am. Med. Dir. Assoc..

[14] Nakatsu N., Sawa R., Misu S., Ueda Y., Ono R. (2015). Reliability and Validity of the Japanese Version of the Simplified Nutritional Appetite Questionnaire in Community-Dwelling Older Adults: Creating Japanese Appetite Questionnaire. Geriatr. Gerontol. Int..

[15] İlhan B., Bahat G., Oren M.M., Kiliç C., Durmazoglu S., Karan M.A. (2018). Reliability and Validity of Turkish Version of the Simplified Nutritional Appetite Questionnaire (SNAQ). J. Nutr. Health Aging.

[16] Akın S., Ozer F.F., Ertürk Zararsız G., Şafak E.D., Mucuk S., Göçer Ş., Mazıcıoğlu M. (2019). Validity of Simplified Nutritional Appetite Questionnaire for Turkish Community-Dwelling Elderly and Determining Cut-off According to Mini Nutritional Assessment. Arch. Gerontol. Geriatr..

[17] Sties S.W., Gonzáles A.I., Viana M.d.S., Brandt R., Bertin R.L., Goldfeder R., Ulbrich A.Z., Andrade A., Carvalho T.d. (2012). Questionário Nutricional Simplificado de Apetite (QNSA) Para Uso Em Programas de Reabilitação Cardiopulmonar e Metabólica. Rev. Bras. Med. Esporte..

[18] Andreae C., Strömberg A., Sawatzky R., Årestedt K. (2015). Psychometric Evaluation of Two Appetite Questionnaires in Patients With Heart Failure. J. Card. Fail..

[19] Yaxley A., Crotty M., Miller M. (2015). Identifying Malnutrition in an Elderly Ambulatory Rehabilitation Population: Agreement between Mini Nutritional Assessment and Validated Screening Tools. Healthcare.

[20] Helfenstein S.F., Uster A., Rühlin M., Pless M., Ballmer P.E., Imoberdorf R. (2016). Are Four Simple Questions Able to Predict Weight Loss in Outpatients With Metastatic Cancer? A Prospective Cohort Study Assessing the Simplified Nutritional Appetite Questionnaire. Nutr. Cancer.

[21] Tokudome Y., Okumura K., Kumagai Y., Hirano H., Kim H., Morishita S., Watanabe Y. (2017). Development of the Japanese Version of the Council on Nutrition Appetite Questionnaire and Its Simplified Versions, and Evaluation of Their Reliability, Validity, and Reproducibility. J. Epidemiol..

[22] Wang T., Shen J. (2018). Usefulness of Simplified Nutritional Appetite Questionnaire (SNAQ) in Appetite Assessment in Elder Patients with Liver Cirrhosis. J. Nutr. Health Aging.

[23] Mohammadi M.R., Akhondzadeh S., Keshavarz S.A., Mostafavi S.-A. (2019). The Characteristics, Reliability and Validity of the Persian Version of Simplified Nutritional Appetite Questionnaire (SNAQ). J. Nutr. Health Aging.

[24] Oh S.Y., Koh S.-J., Baek J.Y., Kwon K.A., Jeung H.-C., Lee K.H., Won Y.-W., Lee H.J. (2019). Validity and Reliability of Korean Version of Simplified Nutritional Appetite Questionnaire in Patients with Advanced Cancer: A Multicenter, Longitudinal Study. Cancer Res. Treat..

[25] Wleklik M., Lisiak M., Andreae C., Uchmanowicz I. (2019). Psychometric Evaluation of Appetite Questionnaires in Elderly Polish Patients with Heart Failure. PPA.

[26] Morley J.E., Malmstrom T.K., Miller D.K. (2012). A Simple Frailty Questionnaire (FRAIL) Predicts Outcomes in Middle Aged African Americans. J. Nutr. Health Aging.

[27] Sahadevan S., Lim P.P., Tan N.J., Chan S.P. (2000). Diagnostic Performance of Two Mental Status Tests in the Older Chinese: Influence of Education and Age on Cut-off Values. Int. J. Geriatr. Psychiatry.

[28] Lim P.P., Ng L.L., Chiam P.C., Ong P.S., Ngui F.T., Sahadevan S. (2000). Validation and Comparison of Three Brief Depression Scales in an Elderly Chinese Population. Int. J. Geriatr. Psychiatry.

[29] Mahoney F.I., Barthel D.W. (1965). Functional Evaluation: The Barthel Index. Md. State Med. J..

[30] Lawton M.P., Brody E.M. (1969). Assessment of Older People: Self-Maintaining and Instrumental Activities of Daily Living. Gerontologist.

[31] Schuling J., de Haan R., Limburg M., Groenier K.H. (1993). The Frenchay Activities Index. Assessment of Functional Status in Stroke Patients. Stroke.

[32] Hurtig-Wennlöf A., Hagströmer M., Olsson L.A. (2010). The International Physical Activity Questionnaire Modified for the Elderly: Aspects of Validity and Feasibility. Public Health Nutr..

[33] Peel C., Sawyer Baker P., Roth D.L., Brown C.J., Brodner E.V., Allman R.M. (2005). Assessing Mobility in Older Adults: The UAB Study of Aging Life-Space Assessment. Phys. Ther..

[34] Pek K., Chew J., Lim J.P., Yew S., Tan C.N., Yeo A., Ding Y.Y., Lim W.S. (2020). Social Frailty Is Independently Associated with Mood, Nutrition, Physical Performance, and Physical Activity: Insights from a Theory-Guided Approach. Int. J. Environ. Res. Public Health.

[35] Fried L.P., Tangen C.M., Walston J., Newman A.B., Hirsch C., Gottdiener J., Seeman T., Tracy R., Kop W.J., Burke G. (2001). Frailty in Older Adults: Evidence for a Phenotype. J. Gerontol. A Biol. Sci. Med. Sci..

[36] Chew J., Tay L., Lim J.P., Leung B.P., Yeo A., Yew S., Ding Y.Y., Lim W.S. (2019). Serum Myostatin and IGF-1 as Gender-Specific Biomarkers of Frailty and Low Muscle Mass in Community-Dwelling Older Adults. J. Nutr. Health Aging.

[37] Guralnik J.M., Ferrucci L., Pieper C.F., Leveille S.G., Markides K.S., Ostir G.V., Studenski S., Berkman L.F., Wallace R.B. (2000). Lower Extremity Function and Subsequent Disability: Consistency across Studies, Predictive Models, and Value of Gait Speed Alone Compared with the Short Physical Performance Battery. J. Gerontol. A Biol. Sci. Med. Sci..

[38] Oh B., Cho B., Choi H.-C., Son K.-Y., Park S.M., Chun S., Cho S.-I. (2014). The Influence of Lower-Extremity Function in Elderly Individuals’ Quality of Life (QOL): An Analysis of the Correlation between SPPB and EQ-5D. Arch. Gerontol. Geriatr..

[39] Chen L.-K., Woo J., Assantachai P., Auyeung T.-W., Chou M.-Y., Iijima K., Jang H.C., Kang L., Kim M., Kim S. (2020). Asian Working Group for Sarcopenia: 2019 Consensus Update on Sarcopenia Diagnosis and Treatment. J. Am. Med. Dir. Assoc..

[40] Guigoz Y., Lauque S., Vellas B.J. (2002). Identifying the Elderly at Risk for Malnutrition. The Mini Nutritional Assessment. Clin. Geriatr. Med..

[41] Bauer J.M. (2013). The MNA in 2013—Still Going Stronger after Almost Twenty Years. J. Nutr. Health Aging.

[42] Vellas B., Villars H., Abellan G., Soto M.E., Rolland Y., Guigoz Y., Morley J.E., Chumlea W., Salva A., Rubenstein L.Z. (2006). Overview of the MNA—Its History and Challenges. J. Nutr. Health Aging.

[43] Wetzel A.P. (2012). Factor Analysis Methods and Validity Evidence: A Review of Instrument Development Across the Medical Education Continuum. Acad. Med..

[44] Covinsky K.E., Martin G.E., Beyth R.J., Justice A.C., Sehgal A.R., Landefeld C.S. (1999). The Relationship Between Clinical Assessments of Nutritional Status and Adverse Outcomes in Older Hospitalized Medical Patients. J. Am. Geriatr. Soc..

[45] Sullivan D.H., Bopp M.M., Roberson P.K. (2002). Protein-Energy Undernutrition and Life-Threatening Complications among the Hospitalized Elderly. J. Gen. Intern. Med..

[46] Wei K., Nyunt M.-S.-Z., Gao Q., Wee S.-L., Yap K.-B., Ng T.-P. (2018). Association of Frailty and Malnutrition With Long-Term Functional and Mortality Outcomes Among Community-Dwelling Older Adults: Results From the Singapore Longitudinal Aging Study 1. JAMA Netw. Open.

[47] Asamane E.A., Greig C.A., Aunger J.A., Thompson J.L. (2019). Perceptions and Factors Influencing Eating Behaviours and Physical Function in Community-Dwelling Ethnically Diverse Older Adults: A Longitudinal Qualitative Study. Nutrients.

[48] Gilbert P.A., Khokhar S. (2008). Changing Dietary Habits of Ethnic Groups in Europe and Implications for Health: Nutrition Reviews©, Vol. 66, No. 4. Nutr. Rev..

[49] Schroll K., Moreiras-Varela O., Schlettwein-Gsell D., Decarli B., de Groot L., van Staveren W. (1997). Cross-Cultural Variations and Changes in Food-Group Intake among Elderly Women in Europe: Results from the Survey in Europe on Nutrition and the Elderly a Concerted Action (SENECA). Am. J. Clin. Nutr..

[50] Herman C.P. (2015). The Social Facilitation of Eating. A Review. Appetite.

[51] Kamphuis C.B., de Bekker-Grob E.W., van Lenthe F.J. (2015). Factors Affecting Food Choices of Older Adults from High and Low Socioeconomic Groups: A Discrete Choice Experiment. Am. J. Clin. Nutr..

[52] Conklin A.I., Maguire E.R., Monsivais P. (2013). Economic Determinants of Diet in Older Adults: Systematic Review. J. Epidemiol. Community Health.

[53] Higgs S., Thomas J. (2016). Social Influences on Eating. Curr. Opin. Behav. Sci..

[54] Vesnaver E., Keller H.H. (2011). Social Influences and Eating Behavior in Later Life: A Review. J. Nutr. Gerontol. Geriatr..

[55] Di Francesco V., Fantin F., Omizzolo F., Residori L., Bissoli L., Bosello O., Zamboni M. (2007). The Anorexia of Aging. Dig. Dis..

[56] Morley J.E., Silver A.J. (1988). Anorexia in the Elderly. Neurobiol. Aging.

[57] Gosnell B.A., Levine A.S., Morley J.E. (1983). The Effects of Aging on Opioid Modulation of Feeding in Rats. Life Sci..

[58] MacIntosh C.G., Morley J.E., Wishart J., Morris H., Jansen J.B.M.J., Horowitz M., Chapman I.M. (2001). Effect of Exogenous Cholecystokinin (CCK)-8 on Food Intake and Plasma CCK, Leptin, and Insulin Concentrations in Older and Young Adults: Evidence for Increased CCK Activity as a Cause of the Anorexia of Aging. J. Clin. Endocrinol. Metab..

[59] Jones K.L., Doran S.M., Hveem K., Bartholomeusz F.D., Morley J.E., Sun W.M., Chatterton B.E., Horowitz M. (1997). Relation between Postprandial Satiation and Antral Area in Normal Subjects. Am. J. Clin. Nutr..

[60] Cornali C., Franzoni S., Frisoni G.B., Trabucchi M. (2005). Anorexia as an Independent Predictor of Mortality. J. Am. Geriatr. Soc..

[61] Landi F., Russo A., Liperoti R., Tosato M., Barillaro C., Pahor M., Bernabei R., Onder G. (2010). Anorexia, Physical Function, and Incident Disability Among the Frail Elderly Population: Results From the IlSIRENTE Study. J. Am. Med. Dir. Assoc..

[62] Morley J.E. (2010). Anorexia, Weight Loss, and Frailty. J. Am. Med. Dir. Assoc..

[63] Morley J.E., Adams E.V. (2015). Rapid Geriatric Assessment. J. Am. Med. Dir. Assoc..

